# Complete genome sequence of *Leadbetterella byssophila* type strain (4M15^T^)

**DOI:** 10.4056/sigs.1413518

**Published:** 2011-03-04

**Authors:** Birte Abt, Hazuki Teshima, Susan Lucas, Alla Lapidus, Tijana Glavina Del Rio, Matt Nolan, Hope Tice, Jan-Fang Cheng, Sam Pitluck, Konstantinos Liolios, Ioanna Pagani, Natalia Ivanova, Konstantinos Mavromatis, Amrita Pati, Roxane Tapia, Cliff Han, Lynne Goodwin, Amy Chen, Krishna Palaniappan, Miriam Land, Loren Hauser, Yun-Juan Chang, Cynthia D. Jeffries, Manfred Rohde, Markus Göker, Brian J. Tindall, John C. Detter, Tanja Woyke, James Bristow, Jonathan A. Eisen, Victor Markowitz, Philip Hugenholtz, Hans-Peter Klenk, Nikos C. Kyrpides

**Affiliations:** 1 DSMZ - German Collection of Microorganisms and Cell Cultures GmbH, Braunschweig, Germany; 2 DOE Joint Genome Institute, Walnut Creek, California, USA; 3 Los Alamos National Laboratory, Bioscience Division, Los Alamos, New Mexico USA; 4 Biological Data Management and Technology Center, Lawrence Berkeley National Laboratory, Berkeley, California, USA; 5 Lawrence Livermore National Laboratory, Livermore, California, USA; 6 HZI – Helmholtz Centre for Infection Research, Braunschweig, Germany; 7 University of California Davis Genome Center, Davis, California, USA; 8 Australian Centre for Ecogenomics, School of Chemistry and Molecular Biosciences, The University of Queensland, Brisbane, Australia

**Keywords:** non-motile, non-sporulating, aerobic, mesophile, Gram-negative, flexirubin, *Cytophagaceae*, GEBA

## Abstract

*Leadbetterella byssophila* Weon *et al*. 2005 is the type species of the genus *Leadbetterella* of the family *Cytophagaceae* in the phylum *Bacteroidetes*. Members of the phylum *Bacteroidetes* are widely distributed in nature, especially in aquatic environments. They are of special interest for their ability to degrade complex biopolymers. *L. byssophila* occupies a rather isolated position in the tree of life and is characterized by its ability to hydrolyze starch and gelatine, but not agar, cellulose or chitin. Here we describe the features of this organism, together with the complete genome sequence, and annotation. *L. byssophila* is already the 16^th^ member of the family *Cytophagaceae* whose genome has been sequenced. The 4,059,653 bp long single replicon genome with its 3,613 protein-coding and 53 RNA genes is part of the *** G****enomic* *** E****ncyclopedia of* *** B****acteria and* *** A****rchaea * project.

## Introduction

Strain 4M15^T^ (= DSM 17132 = JCM 16389 = KACC 11308) is the type strain of the species *Leadbetterella byssophila*, which is the type species of the genus *Leadbetterella*. Currently *L. byssophila* is the only validly named  species in this genus. The type strain was isolated by Weon *et al.* [1] from cotton-waste compost soil used as mushroom cultivation in Suwon, South Korea. *L. byssophila* is described as aerobic, rod shaped and non-motile. The species belongs to the *Cytophaga-Flavobacterium-Bacteroides* (CFB) group, also known as the phylum *Bacteroidetes* [2], which comprises organisms associated with the degradation of complex polysaccharides. The CFB group consists of many bacterial strains isolated from marine environments and hypersaline lakes; but only a few were isolated from other habitats such as soil. Various taxonomic treatments have placed *L. byssophila* either in the family '*Flexibacteraceae*' or the family *Cytophagaceae*. This is most probably due to a number of nomenclatural problems. The family '*Flexibacteraceae*' as outlined in TOBA7.7 [3] would include *Cytophaga hutchinsonii*, which is the type species of the genus *Cytophaga*, which in turn is the type of the family *Cytophagaceae*, a name that may not be replaced by the family name '*Flexibacteraceae*' as long as *Cytophaga hutchinsonii* is one of the included species. A similar problem arises with the placement of *Spirosoma linguale* in the higher taxonomic ranks and has been discussed previously [4]. Here we present a summary classification and a set of features for *L. byssophila* 4M15^T^, together with the description of the complete genomic sequencing and annotation.

## Classification and features

*L. byssophila* 4M15^T^ is very isolated in the tree of life, with no other species allocated to the same genus and with the type strains of the members of the genus *Emticicia* [5,6] sharing the highest degree of 16S rRNA sequence identity (88.3-88.9%) [7], followed by *Sporocytophaga myxococcoides* (85.2%) [8] and *Siphonobacter aquaeclarae* (85.0%) [9]. No other cultured strain belonging to the species or genus has been described. Only one 16S rRNA sequence from a moderately related (95% sequence identity) uncultivated clone, W4S69 (GU560170), identified by Xu and colleagues in pharmaceutical wastewater biofilms, was reported in GenBank. Neither environmental screenings nor genomic surveys produced any sequence that could be linked to the species *L. byssophila* or the genus *Leadbetterella*, indicating that members of the species are not heavily represented in the so far screened habitats (status November 2010).

A representative genomic 16S rRNA sequence of *L. byssophila* 4M15^T^ was compared using NCBI BLAST under default settings (e.g., considering only the high-scoring segment pairs (HSPs) from the best 250 hits) with the most recent release of the Greengenes database [10] and the relative frequencies, weighted by BLAST scores, of taxa and keywords (reduced to their stem [11]) were determined. The five most frequent genera were *Pedobacter* (35.1%), *Flectobacillus* (11.4%), *Leadbetterella* (9.4%), *Algoriphagus* (8.6%) and *Arcicella* (6.8%) (80 hits in total). Regarding the single hit to sequences from members of the species, the average identity within HSPs was 100.0%, whereas the average coverage by HSPs was 97.9%. Among all other species, the one yielding the highest score was '*Kaistomonas ginsengisoli*', which corresponded to an identity of 92.2% and a HSP coverage of 49.8%. The name '*Kaistomonas ginsengisoli*' (strain Gsoil 085, AB245370) is also to be found in a number of publications [12-14], but that has since become the type strain of *Emticicia ginsengisoli* [6].

The highest-scoring environmental sequence was HM238135 ('structure full-scale air pig facility biofilter treating waste gas clone FF 92'), which showed an identity of 93.1% and a HSP coverage of 96.0%. The five most frequent keywords within the labels of environmental samples which yielded hits were 'lake' (4.8%), 'litholog/stream' (4.5%), 'biofilm' (3.3%), 'microbi' (2.9%) and 'site' (2.5%) (170 hits in total). The five most frequent keywords within the labels of environmental samples which yielded hits of a higher score than the highest scoring species were 'soil' (5.1%), 'biofilm/oxid' (5.1%), 'air, biofilt, facil, full-scal, pig, structur, treat, wast' (2.7%), 'forest, ghat, ground, india, mangrov, nich, prokaryt, select, studi, swab, western' (2.7%) and 'cold, spring' (2.6%) (5 hits in total).

**Figure 1:**
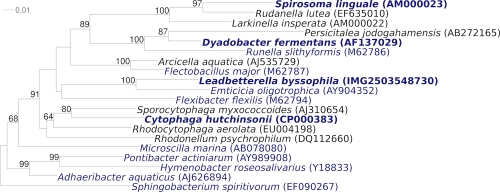
Phylogenetic tree highlighting the position of *L. byssophila* relative to the type strains of the other genera within the family *Cytophagaceae*. The tree was inferred from 1,340 aligned characters [15,16] of the 16S rRNA gene sequence under the maximum likelihood criterion [17] and rooted with the type strain of the closely related family *Sphingobacteriaceae*. The branches are scaled in terms of the expected number of substitutions per site. Numbers above branches are support values from 900 bootstrap replicates [18] if larger than 60%. Lineages with type strain genome sequencing projects registered in GOLD [19] are shown in blue, published genomes in bold [4,20,21].

Figure 1 shows the phylogenetic neighborhood of *L. byssophila* 4M15^T^ in a 16S rRNA based tree. The sequences of the three 16S rRNA gene copies in the genome do not differ from each other, and do not differ from the previously published 16S rRNA sequence (AY854022).

**Figure 2:**
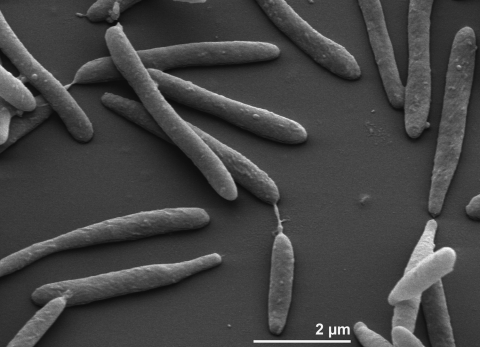
Scanning electron micrograph of *L. byssophila* 4M15^T^

Cells of *L. byssophila* stain Gram-negative and are non-motile, rod shaped with a width of 0.6-0.9 µm and a length of 2-7 µm (Figure 2 and Table 1). Colonies are circular, 1-2 mm in diameter, smooth, light orange, shiny and convex with entire margin when grown on trypticase soy agar. After prolonged incubation colonies become dark orange. Strain 4M15^T^ grows under aerobic conditions at temperatures of 15-45°C and at a pH range of 6.0-8.0. The strain grows in the presence of 1% (w/v) NaCl, but not at 3% NaCl [1]. Growth on carbohydrates (API 20NE) was observed for glucose, arabinose, mannose, N-acetylglucosamine and maltose but not for mannitol. Strain 4M15^T^ was positive for indole production and β-galactosidase, and negative for nitrate reduction and arginine dihydrolase (API 20NE). Enzymatic activity was detected for alkaline phosphatase, leucine arylamidase, valine arylamidase, trypsin, acid phosphatase, naphthol-AS-BI-phosphohydrolase, α-glucosidase, β-glucosidase, N-acetyl-β-glucosaminidase and α-fucosidase; weak enzymatic activity was observed for α-galactosidase and β-galactosidase (API ZYM). *L. byssophila* produces catalase and urease [1]. Whereas starch, gelatin, aesculin and tyrosine are hydrolyzed; agar, casein, cellulose and chitin are not. Strain 4M15^T^ is sensitive to ampicillin, carbencillin, lincomycin, streptomycin and tetracycline and shows resistance to benzylpenicillin, gentamicin, neomycin, oleandomycin and polymyxin B.

**Table 1:** Classification and general features of *L. byssophila* according to the MIGS recommendations [22].

**MIGS ID**	**Property**	**Term**	**Evidence code**
	Current classification	Domain *Bacteria*	TAS [23]
		Phylum *Bacteroidetes*	TAS [24]
		Class *Cytophagia*	TAS [25]
		Order *Sphingobacteriales*	TAS [24]
		Family *Cytophagaceae*	TAS [25, 26]
		Genus *Leadbetterella*	TAS [1]
		Species *Leadbetterella byssophila*	TAS [1]
		Type strain 4M15	TAS [1]
	Gram stain	negative	TAS [1]
	Cell shape	rod-shaped	TAS [1]
	Motility	non-motile	TAS [1]
	Sporulation	non-sporulating	TAS [1]
	Temperature range	mesophile, 15-45°C	TAS [1]
	Optimum temperature	30°C	NAS
	Salinity	1%	TAS [1]
MIGS-22	Oxygen requirement	aerobic	TAS [1]
	Carbon source	glucose, arabinose, mannose, N-acetylglucosamine, and maltose	TAS [1]
	Energy source	chemoorganotrophic	TAS [1]
MIGS-6	Habitat	cotton compost	TAS [1]
MIGS-15	Biotic relationship	free living	NAS
MIGS-14	Pathogenicity	non pathogenic	NAS
	Biosafety level	1	TAS [27]
	Isolation	cotton-waste composts	TAS [1]
MIGS-4	Geographic location	South Korea	TAS [1]
MIGS-5	Sample collection time	2002	NAS
MIGS-4.1	Latitude	not reported	
MIGS-4.2	Longitude	not reported	
MIGS-4.3	Depth	not reported	
MIGS-4.4	Altitude	not reported	

Evidence codes - IDA: Inferred from Direct Assay (first time in publication); TAS: Traceable Author Statement (i.e., a direct report exists in the literature); NAS: Non-traceable Author Statement (i.e., not directly observed for the living, isolated sample, but based on a generally accepted property for the species, or anecdotal evidence). These evidence codes are from of the Gene Ontology project [28]. If the evidence code is IDA, then the property was directly observed by one of the authors or an expert mentioned in the acknowledgements.

### Chemotaxonomy

The fatty acids of strain 4M15^T^ comprise a complex mixture of straight chain saturated and unsaturated acids, together with iso-branched, anteiso-branched and hydroxylated acids. The fatty acids comprise iso-C_15:0_ (24.2%), C_16:1_ω5c (2.8%), C_16:0_ (5.6%), iso-C_15:0_ 3-OH (2.8%), iso-C_17:1_ω9c (1.4%), C_16:0_ 3-OH (2.5%), iso-C_17:0_ 3-OH (10.5%) and the summed feature listed in the MIDI Sherlock system as C_16:1_ ω7c and/or iso-C_15:0_ 2-OH (30.5%) and iso-C_15:0_ 2-OH and/or C_16:1_ ω7c (15.9%). These two peaks probably represent C_16:1_ ω7c (30.5%) and iso-C_15:0_ 2-OH (15.9%) of the fatty acids. The presence of saturated, unsaturated straight chain fatty acids, together with branched chain (largely iso-) normal and 2-OH and 3-OH derivatives is fairly typical of the aerobic members of the *Bacteroidetes*. The major isoprenoid quinone is menaquinone MK-7, a trace amount of menaquinone MK-8 was detected [1]. The polar lipids of *L. byssophila* have not been determined, but for a number of other taxa, including *Rudanella lutea* 5715S-11^T^, *S. linguale* DSM 74^T^, *Larkinella insperata* LMG 22510^T^ and some members of the genus *Sphingobacterium* and *Parapedobacter* [29,30] the presence of phosphatidylethanolamine as the major (diglyceride based) phospholipid and a number of not further characterized lipids and amino lipids provide useful taxonomic and evolutionary markers [31].

## Genome sequencing and annotation

### Genome project history

This organism was selected for sequencing on the basis of its phylogenetic position [32], and is part of the *** G****enomic* *** E****ncyclopedia of* *** B****acteria and* *** A****rchaea * project [33]. The genome project is deposited in the Genomes OnLine Database [19] and the complete genome sequence is deposited in GenBank. Sequencing, finishing and annotation were performed by the DOE Joint Genome Institute (JGI). A summary of the project information is shown in Table 2.

**Table 2:** Genome sequencing project information

**MIGS ID**	**Property**	**Term**
MIGS-31	Finishing quality	Finished
MIGS-28	Libraries used	Genomic libraries: one 454 pyrosequence standard library, one 454 PE library (11.7 kb insert size); one illumina standard library
MIGS-29	Sequencing platforms	454 Titanium, Illumina
MIGS-31.2	Sequencing coverage	71.4 × pyrosequence; 105.0 × Illumina
MIGS-30	Assemblers	Newbler version 2.3-PreRelease-10-21- 2009-gcc-4.1.2-threads, phrap, Velvet
MIGS-32	Gene calling method	Prodigal 1.4, GenePRIMP
	INSDC ID	CP002305
	Genbank Date of Release	November 12, 2010
	GOLD ID	Gc01535
	NCBI project ID	38283
	Database: IMG-GEBA	2503538002
MIGS-13	Source material identifier	DSM 17132
	Project relevance	Tree of Life, GEBA

### Growth conditions and DNA isolation

*L. byssophila* 4M15^T^, DSM 17132, was grown in DSMZ medium 545 (Tryptone soya broth, TSB) [34] at 30°C. DNA was isolated from 0.5-1 g of cell paste using Qiagen Genomic 500 DNA Kit (Qiagen, Hilden, Germany) following the standard protocol as recommended by the manufacturer with modification st/L for cell lysis as described in Wu *et al*. [33]. DNA is available through the DNA bank Network [35,36].

### Genome sequencing and assembly

The genome was sequenced using a combination of Illumina and 454 sequencing platforms. All general aspects of library construction and sequencing can be found at the JGI website [37]. Pyrosequencing reads were assembled using the Newbler assembler version 2.3-PreRelease-10-21-2009-gcc-4.1.2-threads (Roche). The initial Newbler assembly consisting of 73 contigs in one scaffold was converted into a phrap assembly by making fake reads from the consensus, collecting the read pairs in the 454 paired end library. Illumina GAii sequencing data (420.0 Mb) was assembled with Velvet [38] and the consensus sequences were shredded into 1.5 kb overlapped fake reads and assembled together with the 454 data. The 454 draft assembly was based on 237.2 MB 454 draft data and all of the 454 paired end data. Newbler parameters are -consed -a 50 -l 350 -g -m -ml 20.

The Phred/Phrap/Consed software package [39] was used for sequence assembly and quality assessment in the following finishing process. After the shotgun stage, reads were assembled with parallel phrap (High Performance Software, LLC). Possible mis-assemblies were corrected with gapResolution [37], Dupfinisher, or sequencing cloned bridging PCR fragments with subcloning or transposon bombing (Epicentre Biotechnologies, Madison, WI) [40]. Gaps between contigs were closed by editing in Consed, by PCR and by Bubble PCR primer walks (J.-F.Chang, unpublished). A total of 195 additional reactions were necessary to close gaps and to raise the quality of the finished sequence. Illumina reads were also used to correct potential base errors and increase consensus quality using a software Polisher developed at JGI [41]. The error rate of the completed genome sequence is less than 1 in 100,000. Together, the combination of the Illumina and 454 sequencing platforms provided 176.4 × coverage of the genome. Final assembly contains 1,209,137 pyrosequence and 14,794,926 Illumina reads.

### Genome annotation

Genes were identified using Prodigal [42] as part of the Oak Ridge National Laboratory genome annotation pipeline, followed by a round of manual curation using the JGI GenePRIMP pipeline [43]. The predicted CDSs were translated and used to search the National Center for Biotechnology Information (NCBI) nonredundant database, UniProt, TIGRFam, Pfam, PRIAM, KEGG, COG, and InterPro databases. Additional gene prediction analysis and functional annotation was performed within the Integrated Microbial Genomes - Expert Review (IMG-ER) platform [44].

## Genome properties

**Figure 3:**
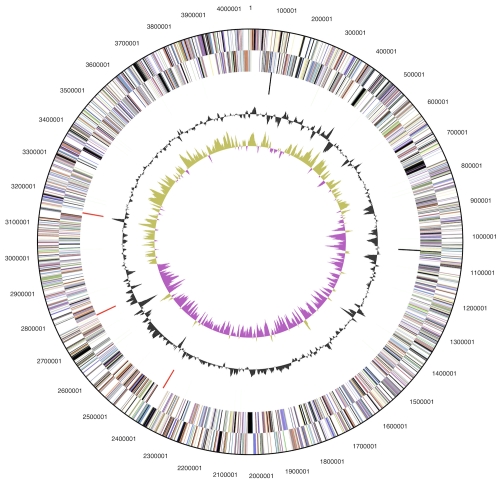
Graphical circular map of the genome. From outside to the center: Genes on forward strand (color by COG categories), Genes on reverse strand (color by COG categories), RNA genes (tRNAs green, rRNAs red, other RNAs black), GC content, GC skew.

The genome is 4,059,653 bp long and comprises one circular chromosome with a 40.4% G+C content (Table 3 and Figure 3). Of the 3,666 genes predicted, 3,613 were protein-coding genes, and 53 RNAs; 148 pseudogenes were also identified. The majority of the protein-coding genes (64.8%) were assigned with a putative function while the remaining ones were annotated as hypothetical proteins. The distribution of genes into COGs functional categories is presented in Table 4.

**Table 3:** Genome Statistics

**Attribute**	Value	% of Total
Genome size (bp)	4,059,653	100.00%
DNA coding region (bp)	3,643,561	89.75%
DNA G+C content (bp)	1,640,653	40.41%
Number of replicons	1	
Extrachromosomal elements	0	
Total genes	3,666	100.00%
RNA genes	53	1.45%
rRNA operons	1	
Protein-coding genes	3,613	98.55%
Pseudo genes	148	4.04%
Genes with function prediction	2,375	64.78%
Genes in paralog clusters	502	13.69%
Genes assigned to COGs	2,353	64.18%
Genes assigned Pfam domains	2,618	71.41%
Genes with signal peptides	985	26.29%
Genes with transmembrane helices	711	19.39%
CRISPR repeats	3	

**Table 4:** Number of genes associated with the general COG functional categories

Code	value	%age	Description
J	152	5.9	Translation, ribosomal structure and biogenesis
A	0	0.0	RNA processing and modification
K	182	7.0	Transcription
L	181	7.0	Replication, recombination and repair
B	1	0.0	Chromatin structure and dynamics
D	23	0.9	Cell cycle control, mitosis and meiosis
Y	0	0.0	Nuclear structure
V	70	2.7	Defense mechanisms
T	142	5.5	Signal transduction mechanisms
M	215	8.3	Cell wall/membrane biogenesis
N	4	0.2	Cell motility
Z	0	0.0	Cytoskeleton
W	0	0.0	Extracellular structures
U	41	1.6	Intracellular trafficking and secretion
O	109	4.2	Posttranslational modification, protein turnover, chaperones
C	142	5.5	Energy production and conversion
G	171	6.6	Carbohydrate transport and metabolism
E	197	7.6	Amino acid transport and metabolism
F	76	2.9	Nucleotide transport and metabolism
H	109	4.2	Coenzyme transport and metabolism
I	90	3.5	Lipid transport and metabolism
P	147	5.7	Inorganic ion transport and metabolism
Q	54	2.1	Secondary metabolites biosynthesis, transport and catabolism
R	321	12.4	General function prediction only
S	164	6.3	Function unknown
-	1,313	35.8	Not in COGs

## Insights from genome sequence

The orange color of *L. byssophila* is due to the presence of flexirubin, a pigment consisting of an ω-phenyloctaenic acid chromophore esterified with resorcinol carrying two hydrocarbon chains. Flexirubins are yellow under neutral pH conditions and become red under alkaline conditions. In *Flavobacterium johnsoniae* a cluster of genes involved in flexirubin synthesis was identified [45], with Fjoh_1102 and Fjoh_1103 having likely roles in the synthesis. Homologs of these genes were also identified in the flexirubin-producing *Bacteroidetes Flavobacterium psychrophilum* and *C. hutchinsonii*, Fjoh_1102 and Fjoh_1103 are similar to *Pseudomonas aurantiaca* *darA* and *darB* which are involved in biosynthesis of the antifungal compound 2-hexyl-5-propyl-alkylresorcinol. In 2009 McBride and colleagues demonstrated the important role of Fjoh_1102 and Fjoh_1103 in flexirubin synthesis by constructing an insertion mutant which was cream colored because of the missing flexirubin [45]. Homologs of Fjoh_1102 and Fjoh_1103 were identified in the genome of *L. byssophila* (Lbys_1508 and Lbys_1509), therefore an important function of these genes in the flexirubin synthesis can be inferred.

Although *L. byssophila* was isolated from cotton waste, which is mainly composed of cellulose, lignin and hemicellulose, the ability to degrade carboxymethylcellulose and filter paper could not be detected; solely D-cellobiose was assimilated [1]. A closer look into the genome sequence of *L. byssophila* revealed the presence of three genes coding cellobiose or cellulose hydrolyzing enzymes: two β-glucosidases belonging to glycoside hydrolase family 3 (Lbys_0274, Lbys_1260) and one endoglucanase belonging to glycoside hydrolase family 5 (Lbys_2254). In order to test whether these endoglucanases actively hydrolyze cellulose we carried out a relatively sensitive assay, using hydroxyethylcellulose with a crosslinked azurin as substrate (AZCL-HEC, Megazyme, Ireland). In this test *L. byssophila* was not able to hydrolyze hydroxyethylcellulose (own unpublished data).This observation conforms with the studies done by Weon and colleagues [1]. The finding of three genes probably coding for α-amylases (Lbys_0590, Lbys_2307, Lbys_2308) is consistent with the observed starch degrading ability of *L. byssophila* [1]. A closer look into the genome sequence revealed four xylan degrading enzymes, three endo-1,4-β-xylanases belonging to glycoside hydrolase family 10 (Lbys_1832, Lbys_2128, Lbys_2331) and one xylan-1,4-β-xylosidase belonging glycoside hydrolase family 43 (Lbys_2333). To date the degradation of xylan by *L. byssophila* was not described, but we could demonstrate the hydrolysis of xylan in a plate assay using xylan with a covalently bound dye as a substrate (remazol brilliant blue-D-xylan, Slovak Academy of Science) (own unpublished data).

*L. byssophila* tested positive for catalase and oxidase [1] The respective genes were identified in the genome sequence. Lbys_1881 encodes a catalase and the genes coding cytochrome C oxidase are localized in the region between Lbys_2190 and Lbys_2195.
